# Distribution and social determinants of overweight and obesity: a cross-sectional study of non-pregnant adult women from the Malawi Demographic and Health Survey (2015-2016)

**DOI:** 10.4178/epih.e2019039

**Published:** 2019-09-27

**Authors:** Leonard Mndala, Abhay Kudale

**Affiliations:** 1 Complete Health Foundation, Lilongwe, Malawi; 2 Interdisciplinary School of Health Sciences, Savitribai Phule Pune University, Pune, India

**Keywords:** Overweight, Obesity, Malawian non-pregnant adult women, Body mass index, Social and demographic determinants, Cross-sectional studies

## Abstract

**OBJECTIVES:**

Hitherto regarded as a public health issue of well-heeled nations, overweight and obesity have emerged as a problem of concern in developing nations. Although social and demographic factors are equally important as proximal lifestyle factors affecting health, their role is neither well researched nor well understood. We conducted a novel study to determine the distribution, prevalence, and social and demographic determinants of overweight/obesity in Malawi.

**METHODS:**

A population-based, quantitative cross-sectional study using data from the Malawi Demographic and Health Survey (2015-2016) was conducted among non-pregnant women aged 18-49 years. A total of 6,443 women were included in the analysis. Overweight/obesity, defined as a body mass index (BMI) ≥25.0 kg/m^2^ , was the main outcome variable. The analysis was done in SPSS version 20.0; after calculating descriptive statistics, bivariate and multivariate logistic regression was conducted to evaluate associations and determine odds.

**RESULTS:**

In total, 16.8% and 6.3% of women were overweight and obese, respectively (p<0.001). Overweight and obesity were more prevalent in urban than in rural areas. The BMI distribution among women varied across different background characteristics. Women from the Ngoni ethnicity were more likely to be overweight/obese than others (adjusted odds ratio [aOR], 1.54; 95% confidence interval [CI], 1.14 to 2.08). Socioeconomic status (SES) and the age of the respondent were highly significant determinants that were strongly associated with being overweight/obese. The richest women were 3 times more likely to be overweight/obese than the poorest (aOR, 3.30; 95% CI, 2.46 to 4.43).

**CONCLUSIONS:**

Overweight and obesity were highly prevalent and significantly associated with increasing SES, age, and being from the Ngoni ethnicity. Holistic interventions should also focus on improving social determinants in order to entirely curb the epidemic.

## INTRODUCTION

In 2016, 2 billion adults aged 18 years and above were overweight, of whom 650 million were obese [1]. Hitherto regarded as a public health issue of well-heeled nations, overweight and obesity have also emerged as a problem of concern in developing low-income and middle-income countries (LMICs) [2]. The last decade has witnessed a noteworthy change in patterns of disease. This change was initially identified by Omran, and it has come to be known as the epidemiologic transition [3]. Tackling overweight and obesity is in accordance with attaining Sustainable Development Goal number 3 (SDG 3) for “Good Health and Wellbeing.”

The World Health Organization (WHO) has prioritized “halting” the rise of obesity by the year 2025 in one of its 9 global voluntary targets to attain success in the fight against noncommunicable diseases (NCDs) [4]. The mounting epidemic in people of all age groups translates into an increasing risk of developing various NCDs, which are responsible for 70% of global mortality [5]. Overweight and obesity are established risk factors for cardiovascular disease (mainly stroke and heart disease), diabetes, musculoskeletal disorders, and many other forms of cancer. Apart from the resultant increased risk of mortality, overweight and obesity push people into poverty globally (especially in LMICs) through associated direct and indirect health care costs [6].

For the past decades, sub-Saharan Africa has been battling against undernutrition and infectious diseases, but in recent years, overweight and obesity and NCDs have put pressure on healthcare systems in this region [7]. The increase in rates of overweight and obesity has occurred more quickly in developing countries, thus translating into a “double-burden of malnutrition,” as overweight and obesity exist side by side with undernutrition. Because overweight and obesity are established risk factors for NCDs, there has also been a “double burden” of persisting infectious diseases and emerging NCDs [8].

Numerous recommendations have been made for global surveillance of modifiable proximal determinants of overweight and obesity, but less attention has been paid to social factors, which in public health are equally important in the holistic understanding of various determinants of health [9]. As health is a social construct, the WHO’s Commission on Social Determinants of Health has set up a comprehensive framework for understanding the different social factors that are associated with health. The framework brings together different aspects of social productivity in health and looks at social context, social stratification, differential exposure and vulnerabilities, and the consequences of ill health [10]. In this framework, behavior, biological, psychosocial factors, and other factors are regarded as social-cohesion factors. In research, although certain behaviors are studied as “lifestyle” factors, the social aspect of this framework stipulates that such factors and even biological factors (such as parity) are embedded within it.

The paucity of disaggregated information on the distribution of overweight and obesity based on various social factors and how such factors influence these conditions in women could mask the seriousness of overweight and obesity in adult non-pregnant women of Malawi. Therefore, research is needed to develop effective, context-specific, and population-based interventions for the prevention of obesity and NCDs. Developing targeted interventions for women based on relevant evidence would also translate into cost-effectiveness. The purpose of this study was to determine the prevalence and social and demographic determinants of overweight and obesity in adult non-pregnant women aged 18-49 years in Malawi using data from the Malawi Demographic and Health Survey (MDHS) 2015-2016.

## MATERIALS AND METHODS

### Study population

This cross-sectional study was conducted using data from the 2015-2016 MDHS. The survey is part of the global Demographic and Health Survey (DHS) program, which assists countries in periodically collecting data to monitor and evaluate populations and health and nutrition programs. The MDHS 2015-2016 was implemented by the National Statistical Office from October 19, 2015 to February 18, 2016. The survey involved interviews with a representative sample of women and men, and collected information on households, data on biomarkers, and anthropometric measurements of women (15-49 years of age) and children under 5 years of age, among other information. As part of this survey, 24,562 women aged 15-49 years were interviewed. We excluded 1,883 women who self-reported that they were pregnant, 3,022 who were below 18 years of age and 13,254 who had missing or flagged height and weight measurements, or for whom anthropometry was not done for other reasons (e.g., refusal). Pregnant women were excluded because pregnancy nullifies results for body mass index (BMI) as an indicator of nutrition status, especially at the population level, and women aged 15-17 years were excluded because they are still undergoing rapid physical changes, meaning that BMI alone is not a good indicator of their nutritional status (in this case overweight and obesity). The result was a final analytical sample of 6,443 adult non-pregnant women aged 18-49 years. Our study involved an exclusive population of women only, because anthropometric data for the MDHS was collected only in women. In addition, globally, more women than men are overweight or obese, and maternal overweight and obesity are associated with numerous health consequences and an emerging cycle of these conditions across multiple generations if left unchecked [11].

### Anthropometric measurements

Height and weight were recoded following the Centers for Disease Control anthropometry protocol [12]. Height was measured in a standing position using standard height boards, while the participant was in a relaxed state. Shoulders were stress-free, and hair pins and all other head buns and ornaments were removed. Each woman was directed to stand straight, with her back against the board, toes apart, and heels together. Height was measured to the nearest 0.1 cm. A calibrated digital scale was used to measure weight. The scale was put on a flat surface and tared to zero. The participant was requested to put on light clothing, and stand straight on the scale, with eyes focused in front. Weight was measured to the nearest 0.1 kg. Height and weight were used to estimate the prevalence of overweight and obesity using BMI (calculated as a continuous variable using the following formula: weight [kg]/height [m]^2^).

### Quantitative variables in the study

The main outcome variable was overweight and obesity (BMI ≥25.0 kg/m^2^). For the regression analysis, BMI was recoded into a binary variable with the categories of not overweight or obese (BMI ≤24.9 kg/m^2^) and overweight or obese (≥25.0 kg/m^2^). For other ancillary analyses, BMI was categorized based on the WHO [13] classes as follows: underweight (<18.5 kg/m^2^), normal (18.5-24.9 kg/m^2^), overweight (25.0-29.9 kg/m^2^), obese (≥30.0 kg/m^2^), pre-obese (25.0-29.9 kg/m^2^), obese class I (30.0-34.9 kg/m^2^), obese class II (35.0-39.9 kg/m^2^), and obese class III (≥40.0 kg/m^2^).

The covariates (social and demographic associates) were presented and defined as follows: age in years (calculated as a continuous variable and stratified into 6 categories: 18-22, 23-27, 28-32, 33-37, 38-42 and ≥43 years), area of residence (rural or urban), marital status (recoded into 6 categories: never in union, married, living with partner, widowed, divorced, and separated), educational status (categorized as no education, primary education, secondary education, and higher education), occupation (recoded and categorized into 8 categories based on the International Standard Classification of Occupations [14]: not working, professional/managerial/technician, clerical/sales, agriculture, household and domestic, services, skilled manual and unskilled manual), socioeconomic status (SES) (indicators of SES were assessed using the wealth index quintile, ranging from first to fifth, which translate to poorest to richest in that order), health insurance (yes or no regardless of the insurance source), parity (number of births in the previous 5 years, categorized as none, 1, or ≥2 births), total number of people living in the household (3 or fewer, 4-7, 8-10, or ≥11 people), current smoking status (does not smoke or currently smokes), contraceptive use (using any form of contraceptive or not using any form of contraceptive, religion (recoded into 5 categories: none, other Christian, Muslim, Catholic, and Protestant), Ethnicity (recoded into 6 categories: others, Tumbuka, Lhomwe, Yao, Ngoni, and Chewa).

### Statistical analysis

The analysis was carried out in SPSS version 20.0 (IBM Corp., Armonk, NY, USA) for descriptive statistics (frequencies and weighted percentages) and binary logistic regression to evaluate associations and determine odds. Frequency was analyzed and presented as unweighted, but percentages were weighted so that the results represent the Malawian adult non-pregnant women population. The prevalence estimates for underweight, normal weight, overweight, and obesity were computed as percentages with the total sample size as the denominator. Furthermore, the prevalence of overweight and obesity and their associations with different categorical background characteristics were analyzed using the chi-square test. Results were considered statistically significant at p-values <0.05. Binary logistic regression was used to determine possible relationships and the direction of the associations between different independent variables and the outcome. Bivariate binary logistic regression yielded crude odds ratios (with 95% confidence intervals [CI]) for membership in the overweight or obese category. This was followed by multivariate logistic analysis to adjust for all the social and demographic determinants of the study (independent variables), thereby generating adjusted odds ratios (aOR) and 95% CIs.

### Ethics statement

Prior to conducting this research, a request for approval to download and use datasets (MDHS 2015-16) was sort from the DHS programme. The programme’s institutional review board granted authorization (AuthLetter_120130). On the part of the data source, DHS maintains good ethical standards in all the surveys it conducts. The MDHS 2015-16 was preceded by a national campaign to raise public awareness of the survey aims and give information about its process. Participation in the survey conducted was completely voluntary and with full autonomy to take part or to reject participation at any point of the survey. No perceived potential harm was encountered on refusing participation. Informed, verbal individual consent was obtained from respondents before conducting the questionnaire or interview. All participants’ information were processed anonymously and labeled with just Identification codes. The survey was supervised and approved by the Ministry of Health.

## RESULTS

### Background characteristics

The background characteristics of the study subjects are presented in Table 1. Of all the participants, 81.5% were from rural areas, 68.0% were married, and 59.0% had completed up to primary-level education. The 18-22 age group accounted for 23.7% of the participants, while the lowest percentage was found in the 38-42 age group (10.9%). With regard to religion, 26.4% of the respondents were Protestants, 17.5% Catholics, 12.6% Muslims, and over a quarter (42.9%) belonged to other forms of Christianity. Furthermore, 23.6% of the subjects were in the fifth wealth index quintile, indicating the highest level of SES, 19.3% were in the poorest (first), 19.2% were in the fourth quintile (rich), 19.1% were poor (second quintile), and 18.8% were in the middle class (third quintile).

### Distribution of body mass index categories across subjects in Malawi

Table 2 presents a summary of the anthropometric measurements of study subjects. The subjects in rural areas were marginally older than those in urban areas (30.00±8.70 years vs. 29.00± 7.80 years, respectively). The mean weight, height, and BMI of women were 56.31±10.40 kg, 1.56±0.06 m, and 23.06±3.94 kg/m^2^, respectively. Differences in background characteristics were significantly associated with BMI, except for region of residence (p<0.001 vs. p=0.056) (Table 3). Overall, 5.7% of the women fell into the <18.5 kg/m^2^ (underweight) category of BMI, with no significant differences by area of residence (5.2% in urban areas vs. 5.8% in rural areas; p=0.046; comparison with an alpha of 0.006) (Table 3). Approximately 16.8% of the respondents fell into the BMI category of 25.0-29.9 kg/m^2^ (pre-obese/overweight). The distribution of overweight was significantly different between urban and rural residents (25.5% in urban areas and 14.8% in rural areas; p<0.001). Overweight was almost equally pronounced in women aged 28-32 years and those aged 33-37 years (21.6% and 21.4%, respectively). Over a quarter (26.1%) of women with a higher level of education and those in the richest wealth quintile (25.8%) were overweight. Overall, 6.3% of women were in the obese (BMI ≥30.0 kg/m^2^) category, with significant differences between rural and urban areas (14.6% in urban areas vs. 4.4% in rural areas; p<0.001). Obesity was more pronounced in women aged 33-37 years (11.6%), those with a higher level of education (15.2%), those who had a professional/managerial/technician occupation (15.9%), and those in the richest SES group (14.6%).

Overall, the prevalence of overweight and obesity among adult women in the study was 16.8% and 6.3%, respectively. In urban areas, 25.5% and 14.6% of the women were overweight and obese, respectively. In contrast, in rural areas, 14.8% and 4.4% of the women were overweight and obese, respectively.

**Figure 1.**
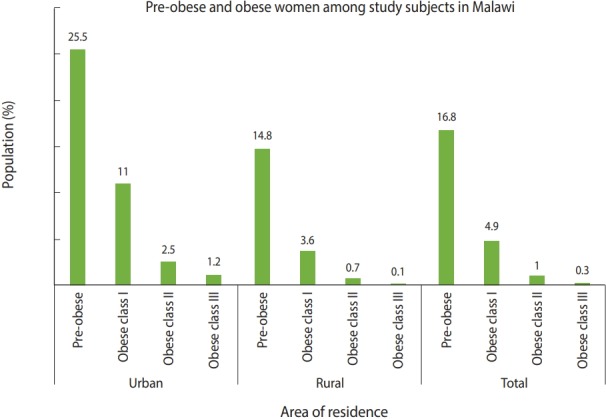
Presents a summary of the prevalence of overweight and obesity by area of residence.

An ancillary categorization of obesity (BMI ≥30.0 kg/m^2^) (Figure 1) by area of residence showed that 4.9% of the respondents were obese class I, 1.0% were obese class II, and 0.3% were obese class III. In urban areas, 11.0%, 2.5%, and 1.2% of the respondents were obese class I, obese class II, and obese class III, respectively. In rural areas, 3.6% of the respondents were obese class I, 0.7% were obese class II, and 0.1% were obese class III. The distribution of pre-obese, obese class I, obese class II, and obese class III women was also considered in reference to SES strata (Table 4). Approximately 11.3% of women in the highest SES stratum were obese class I, and more than a quarter (25.8%) were pre-obese. In the poorest stratum, the prevalence of pre-obese women was 10.9%, marginally higher than in poor women (10.8%).

### Social determinants associated with overweight and obesity among study subjects in Malawi

Table 5 shows the results of binary logistic regression for the predictive probability for membership in the category of overweight or obese. Women who were in the age group of 33-37 years were 4 times more likely to be overweight or obese than those aged between 18 and 22 years (aOR, 3.95; 95% CI, 2.91 to 5.36; p<0.001). The odds of a woman residing in urban area being overweight or obese were 18.0% higher than those of a woman residing in rural area (aOR, 1.18; 95% CI, 0.93 to 1.49). Married women were less likely to be overweight or obese than those who had never been married. The crude odds ratios suggested that the odds of being overweight or obese increased with increasing levels of education, but this was not confirmed in the multivariate analysis. Women from the Ngoni ethnic group were more likely to be overweight or obese than women of other ethnic groups in Malawi (aOR, 1.54; 95% CI, 1.14 to 2.08; p<0.05).

Women in agricultural occupations (self-employed or employee) were less likely to be overweight or obese, which was highly statistically significant (aOR, 0.69; 95% CI, 0.57 to 0.84; p<0.001), while women who were skilled manual workers were more likely to be overweight or obese than those who were not working (aOR, 1.59; 95% CI, 0.96 to 2.64). The risk of being overweight or obese increased with increasing SES, as women belonging to the richest (fifth quintile of the wealth index) group were over 3 times more likely to be overweight or obese than those in the poorest quintile (aOR, 3.30; 95% CI, 2.46 to 4.43). Coverage by any form of health insurance was associated with being overweight or obese, and current smokers were less likely to be overweight or obese. A higher likelihood of being overweight or obese was seen in women residing in households with 8-10 people, as compared to those having 3 or fewer people (aOR, 1.09; 95% CI, 0.81 to 1.46).

## DISCUSSION

Using the MDHS 2015-2016 data, we assessed the overall distribution of BMI (focusing on overweight and obesity) across different social and demographic categories. Furthermore, we determined the different social determinants of overweight and obesity in adult non-pregnant women in Malawi. Different social factors showed significant correlations with overweight and obesity. Overall, the study found that there was a relatively low prevalence of underweight and a relatively high prevalence of overweight and obesity. The combined prevalence of overweight and obesity in adult non-pregnant women of Malawi was estimated to be 23.1%. The results found are comparable with those found in Nigeria (29.2%) [16], but are in contrast with those found in Uganda (11.3%) [17]. The prevalence of overweight and obesity varied across different background characteristics of the women in Malawi. This disparity has also been observed in Nepal [18].

The prevalence of overweight and obesity was higher in urban areas than in rural areas. A high burden of overweight and obesity in women residing in urban areas was also reported in other LMICs, including African countries [19-21]. This tendency could be attributed to rural residents being more actively involved in a less sedentary lifestyle and more laborious activities [22] than urban residents, whose occupations may encourage sedentariness. It should be noted that the high prevalence of overweight and obesity in women may also be due to their physiology, as they tend to deposit more fat than lean mass [23-25].

One of the novel elements of this study is that it further categorized BMIs of ≥25.0 kg/m^2^ into categories of pre-obese, obese class I, obese class II, and obese class III, in order to elucidate clearly the severity of the problem and the need for an immediate response in terms of interventions. Despite the fact that poverty is an established factor in Malawi, there were still some women who were obese class III; this confirms that even the most severe form of obesity is not only a problem of the affluent. Obesity is a morbid state that requires immediate reversal to prevent premature mortality [26].

This study found that SES and age were highly significant determinants of overweight and obesity among women in Malawi. Higher levels of SES were associated with an increased prevalence and likelihood of being overweight or obese in this study, indicating that these women could be susceptible to NCDs associated with being overweight or obese. These findings could be explained by the availability of affordable, energy-dense foods due to urban sprawl, as well as reduced physical activity resulting in a sedentary lifestyle, as observed in a study in Kenya where the women who were most sedentary were in the highest income group [27], and in other studies finding that high-income households purchased foods in bulk and were more likely to overconsume the food [28]. These findings differ from those reported in high-income countries, people with a lower SES were more likely to be overweight or obese due to their tendency to purchase low-quality foods [29]. Multiple studies have indicated that overweight and obesity in women tend to increase with age [30,31]. Similarly, in our study, the odds of being overweight or obese from the ages of 30 years and above were almost 4 times as high compared to women in the 18-22-year-old age category. Starting at the age of 30 years, anecdotal evidence in developing countries shows that different family transitions happen, and women tend to shift various household roles to their children, making themselves more sedentary. In addition, studies have shown that as some women grow old, they tend to express less willingness to reduce weight irrespective of their health status [32]. In resource-restricted healthcare systems like that of Malawi, interventions on curbing overweight and obesity can therefore be prioritized for such age groups.

With regard to area of residence, women who were residing in urban areas were more likely to be overweight or obese. An urban environment is usually associated with an improved economic status of households; households with a high income tend to purchase food in bulk, spending more on both healthy and less healthy foods [32]. This study had some unique key findings. For example, in Malawi, women who belonged to the Ngoni ethnic group were found to have a 54.0% higher risk of being overweight or obese as compared to others (aOR, 1.54; 95% CI, 1.14 to 2.08; p<0.05). Evidence shows that the Ngoni people live a life of hefty meat consumption and alcohol drinking, and chiefly perceive themselves as living a ‘healthy satisfactory life’ [33]. The Ngoni people of Malawi are originally from South Africa and share their roots with the Zulu people, who in contrast to the public health notion that overweight and obesity are unhealthy-view plump women as “healthy” [34].

This study had some limitations. It was cross-sectional in nature and therefore cannot be used to infer causality. However, the motivation was to describe the distribution of overweight and obesity and provide clues regarding potential associations that can be further explored using robust study designs. Despite the essential limitations of BMI as a measure of weight status, it remains the most widely used measure for assessing weight status at the population level. We recognize the importance of measuring proximate factors for overweight and obesity such as physical activity and dietary intake; however, this was beyond the scope of this study and the relevant information was not available in the MDHS data.

Despite its limitations, the main strength of this study is its large sample size and the random clustering sampling method used to recruit participants in the survey, which made it a good representation of adult non-pregnant women in Malawi. The quality of the data is assured as the DHS uses well-trained field personnel, a standardized protocol, and validated tools in the data collection process. The inclusion criteria employed in this study were demanding and further strengthened the quality of the findings. This study also employed an analytical plan that stratified the results based on background characteristics, and also carefully controlled for confounding through a multivariate analysis. These results, therefore, present disaggregated information that is generalizable.

In conclusion, this study showed a high prevalence of overweight and obesity among adult non-pregnant women in Malawi. Overweight and obesity were closely associated with different social and demographic determinants, with factors such as age and SES increasing the risk of developing overweight and obesity by about 4 times in women. This study affirms that the roles of social determinants of overweight and obesity need to be mapped for better evidence-based prioritization of interventions and synergistic efforts to curb the epidemic. The time has come to prevent and control overweight and obesity.

## Figures and Tables

**Table 1.** Background characteristics of the study subjects (n=6,443)

Characteristics	n (%)
Age (yr)	
18-22	1,530 (23.7)
23-27	1,316 (20.5)
28-32	1,179 (18.4)
33-37	969 (15.0)
38-42	711 (10.9)
≥43	738 (11.5)
Region	
Northern	1,230 (11.3)
Central	2,201 (42.5)
Southern	3,012 (46.2)
Residence	
Urban	1,401 (18.5)
Rural	5,042 (81.5)
Marital status	
Never in union/single	783 (11.6)
Married	4,318 (68.0)
Living with partner	324 (4.7)
Widowed	239 (3.5)
Divorced	380 (6.1)
Separated	399 (6.0)
Education	
None	856 (13.8)
Primary	3,726 (59.0)
Secondary	1,655 (23.6)
Higher	206 (3.6)
Religion	
Catholic	1,110 (17.5)
Protestant	1,817 (26.4)
Other Christian	2,766 (42.9)
Muslim	722 (12.6)
None	28 (0.5)
Ethnicity	
Chewa	1,922 (34.1)
Tumbuka	658 (8.9)
Lhomwe	1,197 (19.8)
Yao	747 (13.7)
Ngoni	813 (11.8)
Others	1,106 (11.8)
Occupation	
Professional/technician/managerial	392 (5.5)
Clerical/sales	408 (6.2)
Agriculture (self/employee)	2,574 (41.3)
Household and domestic	92 (1.8)
Services	70 (1.4)
Skilled manual	113 (1.7)
Unskilled manual	911 (14.1)
Not working	1,883 (28.1)
Parity in previous 5 yr	
No	2,395 (36.7)
1	2,887 (45.1)
≥2	1,161 (18.2)
Covered by health insurance	
No	6,343 (98.3)
Yes	100 (1.7)
Contraceptive use (of any form)	
Not using	2,861 (44.2)
Using	3,528 (55.8)
Self-reported menopause status	
Not in menopause	6,262 (97.2)
In menopause	181 (2.8)
Current smoking status	
Does not smoke	6,392 (99.3)
Smokes	51 (0.7)
Who makes decision concerning respondent’s health care	
Others	5 (0.1)
Respondent alone	890 (18.9)
Respondent and husband/partner	2,375 (50.5)
Husband/partner alone	1,351 (30.1)
Someone else	21 (0.4)
SES quintiles	
First (poorest)	1,097 (19.3)
Second	1,195 (19.1)
Third	1,186 (18.8)
Fourth	1,282 (19.2)
Fifth (richest)	1,683 (23.6)

SES, socioeconomic status.

**Table 2.** Age, weight, height, and BMI of subjects by area of residence

Areas	Age (yr)	Weight (kg)	Height (m)	BMI (kg/m^2^)
Urban	29.00±7.80	61.43±13.01	1.57±0.06	24.80±4.91
Rural	30.00±8.70	55.12±9.35	1.56±0.06	22.66±3.60
Residence combined	30.22±8.60	56.31±10.40	1.56±0.06	23.06±3.94

Values are presented as mean±standard deviation. BMI, body mass index.

**Table 3.** Distribution of BMI categories among subjects in Malawi

	BMI (kg/m^2^)				
	<18.50 (underweight)	18.50-24.99 (normal weight)	25.00-29.99 (overweight)	≥30.00 (obese)	p-value^1^
Age (yr)					<0.001
18-22	6.3	82.2	10.6	0.9	
23-27	4.7	76.8	13.8	4.7	
28-32	5.7	65.0	21.6	7.7	
33-37	5.6	61.3	21.4	11.6	
38-42	6.2	67.2	19.0	7.6	
≥43	5.8	65.3	19.0	9.9	
Region					0.056
Northern	5.3	67.9	20.3	6.5	
Central	5.4	72.0	15.8	6.8	
Southern	6.1	71.3	16.8	5.8	
Residence					<0.001
Urban	5.2	54.7	25.5	14.6	
Rural	5.8	75.0	14.8	4.4	
p-value^2^	0.046		<0.001	<0.001	
Marital status					<0.001
Never in union	7.8	73.9	15.4	3.0	
Married	5.2	70.6	16.9	7.4	
Living with partner	4.3	76.2	17.2	2.3	
Widowed	6.3	64.4	20.7	8.6	
Divorced	7.7	68.3	19.6	4.4	
Separated	6.3	76.6	13	4.2	
Education					<0.001
None	6.5	70.1	18.3	5.1	
Primary	5.5	75.0	14.6	4.8	
Secondary	6.2	64.7	19.9	9.2	
Higher	1.7	57.0	26.1	15.2	
Religion					<0.001
None	12.5	62.5	15.6	9.4	
Catholic	5.4	71.1	18.6	4.9	
Protestant	3.9	69.8	19.4	6.9	
Other Christian	6.4	71.9	15.3	6.4	
Muslim	7.2	72.5	13.8	6.6	
Ethnicity					<0.001
Others	5.2	69.5	17.3	8.1	
Chewa	5.6	74.4	15.1	4.8	
Tumbuka	4.2	68.4	20.3	7.1	
Lhomwe	6.3	72.0	15.9	5.7	
Yao	6.1	71.1	14.9	7.9	
Ngoni	5.8	64.6	22.1	7.4	
Occupation					<0.001
Not working	5.9	71.0	17.7	5.5	
Professional/mana- gerial/technician	3.7	53.3	27.2	15.9	
Clerical/sales	4.3	63.5	20.5	11.6	
Agriculture (self/ employee)	5.7	78	12.9	3.4	
Household and domestic	0.9	65.5	26.5	7.1	
Services	8.0	55.7	29.5	6.8	
Skilled manual	3.7	57.0	27.1	12.1	
Unskilled manual	7.2	66.1	17.0	9.6	
SES quintile					<0.001
First (poorest)	7.1	80.1	10.9	1.9	
Second	5.2	80.8	10.8	3.3	
Third	5.2	75.7	15.5	3.6	
Fourth	6.2	68.7	18.8	6.3	
Fifth (richest)	4.9	54.7	25.8	14.6	

Values are presented as %. BMI, body mass index; SES, socioeconomic status.

1 Pearson chi-square test.

2 Met the assumption for Bonferroni correction for within-column probabilities (alpha=0.006): multiple-wise analysis is limited, hence not inflating the ‘family’ (type I) error [15].

**Table 4.** Pre-obese/overweight, class I, class II, and class III obese women across socioeconomic status (SES) strata in Malawi

SES quintiles	Body mass index (kg/m^2^)				
	Pre-obese (25.00-29.99)	Obese class I (30.00-34.99)	Obese class II (35.00-39.99)	Obese class III (≥40.00)	p-value^1^
First (poorest)	106 (10.9)	13 (1.3)	5 (0.5)	-	<0.001
Second	136 (10.8)	30 (3.0)	3 (0.2)	1 (0.1)	
Third	186 (15.5)	32 (2.7)	11 (0.8)	-	
Fourth	255 (18.8)	62 (5.0)	11 (0.9)	6 (0.4)	
Fifth (richest)	439 (25.8)	201 (11.3)	39 (2.4)	21 (0.9)	
Total	1,122 (16.8)	338 (4.9)	69 (1.0)	28 (0.3)	

Values are presented as frequency number (%).

1 Pearson chi-square test.

**Table 5.** Logistic regression for determinants of overweight/obesity among women in Malawi

Variables	Crude OR (95% CI)	Adjusted OR (95% CI)
Age (yr)		
18-22	1.00 (reference)	1.00 (reference)
23-27	1.74 (1.41, 2.14)	1.53 (1.14, 2.06)
28-32	3.17 (2.59, 3.88)	3.06 (2.28, 4.10)
33-37	3.77 (3.06, 4.63)	3.95 (2.91, 5.36)
38-42	2.76 (2.20, 3.48)	3.02 (2.15, 4.24)
≥43	3.10 (2.48, 3.88)	3.26 (2.29, 4.64)
Region		
Northern	1.00 (reference)	1.00 (reference)
Central	0.80 (0.66, 0.97)	0.89 (0.65, 1.21)
Southern	0.79 (0.66, 0.97)	0.82 (0.60, 1.12)
Residence		
Rural	1.00 (reference)	1.00 (reference)
Urban	2.82 (2.47, 3.23)	1.18 (0.93, 1.49)
Marital Status		
Never in union/single	1.00 (reference)	1.00 (reference)
Married	1.43 (1.17, 1.74)	0.93 (0.68, 1.27)
Living with partner	1.07 (0.76, 1.50)	-
Widowed	1.86 (1.32, 2.63)	-
Divorced	1.42 (1.05, 1.91)	-
Separated	0.94 (0.68, 1.29)	-
Education		
None	1.00 (reference)	1.00 (reference)
Primary	0.79 (0.66, 0.94)	0.77 (0.62, 0.96)
Secondary	1.34 (1.11, 1.63)	0.91 (0.68, 1.21)
Higher	2.30 (1.69, 3.11)	0.97 (0.58, 1.64)
Religion		
None	1.00 (reference)	1.00 (reference)
Other Christian	0.79 (0.36, 1.75)	0.84 (0.32, 2.17)
Muslim	0.72 (0.32, 1.64)	0.81 (0.30, 2.21)
Catholic	0.87 (0.39, 1.95)	0.81 (0.31, 2.13)
Protestant	1.02 (0.46, 2.26)	0.97 (0.37, 2.55)
Ethnicity		
Others	1.00 (reference)	1.00 (reference)
Tumbuka	1.11 (0.87, 1.43)	0.99 (0.69, 1.40)
Lhomwe	0.81 (0.66, 1.01)	1.10 (0.83, 1.47)
Yao	0.87 (0.69, 1.09)	1.37 (0.95, 1.98)
Ngoni	1.24 (0.99, 1.56)	1.54 (1.14, 2.08)
Chewa	0.73 (0.60, 0.89)	1.08 (0.81, 1.44)
Occupation		
Not working	1.00 (reference)	1.00 (reference)
Professional/technician/managerial	2.49 (1.97, 3.16)	0.99 (0.71, 1.39)
Clerical/sales	1.56 (1.23, 1.99)	1.01 (0.74, 1.37)
Agriculture (self/employee)	0.65 (0.56, 0.75)	0.69 (0.57, 0.84)
Household and domestic	1.68 (1.12, 2.52)	1.28 (0.63, 2.60)
Services	1.85 (1.18, 2.90)	1.22 (0.64, 2.34)
Skilled manual	2.10 (1.40, 3.15)	1.59 (0.96, 2.64)
Unskilled manual	1.21 (1.00, 1.45)	1.07 (0.85, 1.36)
Parity in previous 5 yr		
No	1.00 (reference)	1.00 (reference)
1	0.73 (0.64, 0.83)	0.95 (0.79, 1.15)
≥2	0.47 (0.39, 0.56)	0.72 (0.56, 0.93)
Covered by health insurance		
No	1.00 (reference)	1.00 (reference)
Yes	2.48 (1.68, 3.65)	1.28 (0.71, 2.29)
Contraceptive use (of any form)		
No	1.00 (reference)	1.00 (reference)
Yes	0.91 (0.81, 1.03)	0.95 (0.81, 1.11)
Self-reported menopause status		
Not in menopause	1.00 (reference)	1.00 (reference)
In menopause	0.88 (0.61, 1.27)	0.75 (0.48, 1.17)
Current smoking status		
Not smoking	1.00 (reference)	1.00 (reference)
Smoking	0.82 (0.39, 1.72)	0.92 (0.41, 2.09)
Who makes decision concerning respondent’s health care		
Others	1.00 (reference)	1.00 (reference)
Respondent alone	0.62 (0.09, 3.99)	0.33 (0.05, 2.33)
Respondent and husband/partner	0.68 (0.11, 4.29)	0.37 (0.05, 2.63)
Respondent and other person	0.49 (0.08, 3.09)	0.32 (0.05, 2.23)
Husband/partner alone	0.77 (0.09, 6.32)	0.63 (0.07, 5.98)
Total household members (n)		
≤3	1.00 (reference)	1.00 (reference)
4-7	1.03 (0.89, 1.19)	0.82 (0.66, 1.02)
8-10	1.23 (0.99, 1.51)	1.09 (0.81, 1.46)
≥11	1.12 (0.71, 1.77)	0.91 (0.48, 1.71)
SES quintiles		
First (poorest)	1.00 (reference)	1.00 (reference)
Second	1.12 (0.89, 1.42)	1.08 (0.82, 1.43)
Third	1.61 (1.29, 2.01)	1.31 (1.00, 1.71)
Fourth	2.29 (1.85, 2.83)	1.67 (1.28, 2.18)
Fifth (richest)	4.64 (3.81,5.65)	3.30 (2.46, 4.43)

For the adjusted ORs, multivariate logistic regression was done, adjusting for all other covariates to address possible confounding; For crude ORs, univariate logistic regression was done. ORs, odd ratios; CI, confidence interval; SES, socioeconomic status.

## References

[1] World Health Organization (2018). Overweight and obesity. https://www.who.int/news-room/fact-sheets/detail/obesity-and-overweight.

[2] Barakat-Haddad C, Saeed U, Elliott S (2017). A longitudinal cohort study examining determinants of overweight and obesity in adulthood. Can J Public Health.

[3] Omran AR (1971). The epidemiologic transition. A theory of the epidemiology of population change. Milbank Mem Fund Q.

[4] Popkin BM, Adair LS, Ng SW (2012). Global nutrition transition and the pandemic of obesity in developing countries. Nutr Rev.

[5] World Health Organization (2015). Global status report on non-communicable diseases 2014. https://apps.who.int/iris/bitstream/handle/10665/148114/9789241564854_eng.pdf?sequence=1.

[6] Dee A, Kearns K, O’Neill C, Sharp L, Staines A, O’Dwyer V (2014). The direct and indirect costs of both overweight and obesity: a systematic review. BMC Res Notes.

[7] Agyemang C, Boatemaa S, Frempong GA, Aikins A, Ahima RS (2016). Obesity in sub-Saharan Africa. Metabolic syndrome.

[8] Steyn NP, Ne JH, Parker WA, Ayah R, Mbithe D (2011). Dietary, social, and environmental determinants of obesity in Kenyan women. Scand J Public Health.

[9] Micklesfield LK, Lambert EV, Hume DJ, Chantler S, Pienaar PR, Dickie K (2013). Socio-cultural, environmental and behavioural determinants of obesity in black South African women. Cardiovasc J Afr.

[10] World Health Organization (2010). A conceptual framework for action on the social determinants of health: debates, policy & practice, case studies. http://apps.who.int/iris/bitstream/10665/44489/1/9789241500852_eng.pdf.

[11] Meehan S, Beck CR, Mair-Jenkins J, Leonardi-Bee J, Puleston R (2014). Maternal obesity and infant mortality: a meta-analysis. Pediatrics.

[12] Centers for Disease Control and Prevention (2007). National health and nutrition examination survey (NHANES). https://www.cdc.gov/nchs/data/nhanes/nhanes_07_08/manual_an.pdf.

[13] World Health Organization (2000). Obesity: preventing and managing the global epidemic. https://www.who.int/nutrition/publications/obesity/WHO_TRS_894/en/.

[14] International Labour Office (2016). International standard classification of occupations: structure, group definitions and correspondence tables. https://www.ilo.org/wcmsp5/groups/public/@dgreports/@dcomm/@publ/documents/publication/wcms_172572.pdf.

[15] Nakagawa S (2004). A farewell to Bonferroni: the problems of low statistical power and publication bias. Behav Ecol.

[16] Adebayo RA, Balogun MO, Adedoyin RA, Obashoro-John OA, Bisiriyu LA, Abiodun OO (2014). Prevalence and pattern of overweight and obesity in three rural communities in southwest Nigeria. Diabetes Metab Syndr Obes.

[17] Maher D, Waswa L, Baisley K, Karabarinde A, Unwin N, Grosskurth H (2011). Distribution of hyperglycaemia and related cardiovascular disease risk factors in low-income countries: a cross-sectional population-based survey in rural Uganda. Int J Epidemiol.

[18] Al Kibria GM (2019). Prevalence and factors affecting underweight, overweight and obesity using Asian and World Health Organization cutoffs among adults in Nepal: analysis of the Demographic and Health Survey 2016. Obes Res Clin Pract.

[19] Subramanian SV, Perkins JM, Özaltin E, Davey Smith G (2011). Weight of nations: a socioeconomic analysis of women in low- to middle-income countries. Am J Clin Nutr.

[20] Atek M, Traissac P, El Ati J, Laid Y, Aounallah-Skhiri H, Eymard-Duvernay S (2013). Obesity and association with area of residence, gender and socio-economic factors in Algerian and Tunisian adults. PLoS One.

[21] Letamo G (2011). The prevalence of, and factors associated with, overweight and obesity in Botswana. J Biosoc Sci.

[22] Sola AO, Steven AO, Kayode JA, Olayinka AO (2011). Underweight, overweight and obesity in adults Nigerians living in rural and urban communities of Benue State. Ann Afr Med.

[23] Amegah AK, Lumor S, Vidogo F (2011). Prevalence and determinants of overweight and obesity in adult residents of Cape Coast, Ghana: a hospital-based study. Afr J Food Agric Nutr Dev.

[24] Mogre V, Mwinlenaa PP, Oladele J, Amalba A (2012). Impact of physical activity levels and diet on central obesity among civil servants in Tamale metropolis. J Med Biomed Sci.

[25] Pobee RA, Owusu WB, Plahar WA (2013). The prevalence of obesity among female teachers of child-bearing age in Ghana. Afr J Food Agric Nutr Dev.

[26] Dobbs R, Sawers C, Thompson F, Manyika J, Woetzel JR, Child P (2014). Overcoming obesity: an initial economic analysis. Executive summary. https://www.mckinsey.com/industries/healthcare-systems-and-services/our-insights/how-the-world-could-better-fight-obesity.

[27] Mbochi RW, Kuria E, Kimiywe J, Ochola S, Steyn NP (2012). Predictors of overweight and obesity in adult women in Nairobi Province, Kenya. BMC Public Health.

[28] French SA, Wall M, Mitchell NR (2010). Household income difference in food sources and food items purchased. Int J Behav Nutr Phys Act.

[29] Rolls BJ (2009). The relationship between dietary energy density and energy intake. Physiol Behav.

[30] El-Hazmi M, Warsy A (2002). Relationship between age and the prevalence of obesity and overweight in Saudi population. Bahrain Med Bull.

[31] Dake FA, Tawiah EO, Badasu DM (2011). Sociodemographic correlates of obesity among Ghanaian women. Public Health Nutr.

[32] Duda RB, Darko R, Seffah J, Adanu RM, Anarfi JK, Hill AG (2007). Prevalence of obesity in women of Accra, Ghana. Afr J Health Sci.

[33] Yaya S, Ghosh S, Ghose B (2019). Subjective happiness, health and quality of life and their sociocultural correlates among younger population in Malawi. Soc Sci.

[34] Ogana W (2014). Food decisions and cultural perceptions of overweight and obesity: the case of Zulu women in Durban, South Africa. http://ukzn-dspace.ukzn.ac.za/bitstream/handle/10413/12719/Ogana_Winifred_2014.pdf?sequence=1&isAllowed=y.

